# Community-Acquired Methicillin-Resistant *Staphylococcus aureus* in Institutionalized Adults with Developmental Disabilities^1^

**DOI:** 10.3201/eid0809.020300

**Published:** 2002-09

**Authors:** Abraham Borer, Jacob Gilad, Pablo Yagupsky, Nechama Peled, Nurith Porat, Ronit Trefler, Hannah Shprecher-Levy, Klaris Riesenberg, Miriam Shipman, Francisc Schlaeffer

**Affiliations:** * Soroka University Medical Center and the Ben-Gurion University of the Negev, Beer-Sheva, Israel; † Carmel Medical Center, Haifa, Israel

## Abstract

Methicillin-resistant *Staphylococcus aureus* (MRSA) has recently been reported to emerge in the community setting. We describe the investigation and control of a community-acquired outbreak of MRSA skin infections in a closed community of institutionalized adults with developmental disabilities. In a 9-month period in 1997, 20 (71%) of 28 residents had 73 infectious episodes. Of the cultures, 60% and 32% obtained from residents and personnel, respectively, grew *S. aureus*; 96% and 27% were MRSA. All isolates were genetically related by pulsed-field gel electrophoresis and belonged to a phage type not previously described in the region. No known risk factors for MRSA acquisition were found. However, 58 antibiotic courses had been administered to 16 residents during the preceding 9 months. Infection control measures, antibiotic restriction, and appropriate therapy resulted in successful termination of this outbreak. Selective antibiotic pressure may result in the emergence, persistence, and dissemination of MRSA strains, causing prolonged disease.

Methicillin-resistant *Staphylococcus aureus* (MRSA) poses a therapeutic challenge in acute-care settings (1–4), as well as long-term skilled-nursing facilities (5–8). Recently, MRSA has also been detected in the community more often. The terms and definitions related to community-acquired MRSA remain controversial, and the “community” as a milieu for MRSA acquisition cannot be implicated with a high degree of certainty. Most studies have defined community acquisition as growth within 48–72 hours after hospital admission (9–11), which does not rule out nosocomial acquisition. Patients thought to have acquired MRSA in the community carry risk factors implicated in nosocomial acquisition (12–16).

Outbreaks of community-acquired MRSA infection are extremely rare (17–19). During 1997, we investigated an outbreak of skin and soft-tissue infection involving MRSA in a closed community of institutionalized adults with developmental disabilities. MRSA emerged and disseminated in this setting as a result of an extreme selective pressure exacerbated by heavy and continuous use of ineffective antimicrobial drugs. That such selective pressure was sufficient to promote MRSA emergence in the community underlines the threat associated with current antibiotic prescribing practices in the community.

## Materials and Methods

### Outbreak Setting

The outbreak occurred in a facility for persons with developmental disabilities, located in the Negev, southern Israel. The facility consists of 283 residents living in nine buildings and confined to the institution. Residents are independent with regard to activities of daily living, with minimal contact between residents of different buildings. Staff consists of 120 personnel who work exclusively in the institution and are assigned to specific buildings. Medical attention is provided by an institutional clinic. The outbreak involved a single building (number 15) inhabited by 28 residents and attended by 34 personnel.

### Epidemiologic Investigation

The outbreak investigation began in December 1997, according to the principles of the Declaration of Helsinki. Informed consent was obtained from personnel, and consent for including residents was obtained from legal guardians and the Ministry of Health.

Information was reviewed regarding possible host risk factors (20), including age, sex, diabetes mellitus, malignancy, coronary disease, chronic lung, hepatic or renal diseases, nephrotic syndrome, congestive heart failure, obesity, debilitating conditions, and pressure sores, as well as therapeutic risk factors such as urinary catheters, nasogastric tubes, and other indwelling devices, steroid treatment and antibiotic therapy prescribed during 12 months preceding the outbreak. Admissions to any acute-care facility during the previous 5 years were recorded.

 To confirm clustering and identify common sources of transmission, all 28 residents from building 15 were screened for both methicillin-sensitive *S. aureus* (MSSA) and MRSA carriage in both anterior nares, perineum, and secreting lesions. Nare and exudate cultures were obtained from all personnel in contact with the residents (34 persons). Additionally, nares cultures were randomly obtained from one-fifth of all residents (50 persons) residing in other buildings at the institution.

### Laboratory Investigation

Cultures were obtained by rotating a moistened swab in both nares, perineum area, and secreting lesions and were processed at the Clinical Microbiology Laboratory of the Soroka University Medical Center. Identification of *S. aureus* was performed by routine methods. Methicillin resistance was determined by using a 1-μg oxacillin disk. Susceptibility to erythromycin, clindamycin, cefuroxime, ceftriaxone, ciprofloxacin, gentamicin, fusidic acid, and vancomycin was determined by using the disc-diffusion method. Mupirocin resistance (MIC>256 mg/L) was determined by E-test (AB Biodisk, Solna, Sweden).

Bacteriophage typing at routine test dilutions was performed at a national reference laboratory, Rabin Medical Center, Petah-Tikva, Israel. Methicillin resistance was confirmed by polymerase chain reaction (PCR) for the *mecA* gene (21). Pulsed-field gel DNA electrophoresis (PFGE) for the determination of genetic relatedness was generated by digestion with the restriction endonuclease *Sma*I as described elsewhere (22), and the banding pattern was interpreted according to current consensus (23).

### Intervention

Management of the outbreak was carried out by the four-phase approach of Wenzel et al. (24), with modifications related to the setting under investigation. Basic epidemiologic measures, infection control measures, and isolation precautions were instituted, including glove use during personnel-resident contact, hand washing with 4% chlorhexidine after glove removal, reserving personal washcloths and towels for each resident, bathing daily with 4% chlorhexidine-containing soap, and changing towels, clothing, and bed sheets daily. Draining lesions were covered at all times with sterile dressings, which were promptly discarded after removal.

Treatment to eliminate nasal carriage in culture-positive persons was given after randomization, by using either intranasal mupirocin calcium 2% ointment (Bactroban, Glaxo SmithKline, Philadelphia, PA) or sodium fusidate 2% ointment (Fucidin, Leo Pharmaceutical, Ballerup, Denmark), twice a day for a week. Spontaneous or surgically drained lesions were treated with the same topical antibiotic used intranasally. Systemic therapy with oral fusidic acid 500 mg twice a day (Fucidin, Leo Pharmaceutical) was reserved only for lesions surrounded by cellulitis, located around the mid-face, or in presence of systemic symptoms or signs. To limit antibiotic use, therapy other than the above was not allowed. This phase was supervised by infectious-disease specialists. Thereafter, infection control was supervised weekly by an infection control nurse and every 3 weeks by an infectious-disease specialist.

In the implicated building, follow-up cultures were obtained from all residents and personnel 1 week as well as 1 month after intervention. After 2 additional weeks, repeat cultures were obtained only from those with previous positive culture. Two years later, in March 2000, nares cultures were obtained from of all residents in order to assess the prevalence of persistent carriage.

### Statistical Analysis

Statistical analysis was performed with the Epi-Info software (Version 6.03; 1996, Centers for Disease Control and Prevention, Atlanta, GA), using the chi-square and Fisher’s exact tests as appropriate. A p value of <0.05 was considered statistically significant.

## Results

### Outbreak Description

During mid-1997, an increasing number of skin and soft-tissue infections in residents of a single building were recognized by the staff. No cases were diagnosed in residents in other buildings or the remaining staff. The initial case involved an uncomplicated furuncle in a patient with dermatitis. From March 1, 1997, to December 31, 1997, 60 patient visits related to skin, soft-tissue, ear, and eye infections were recorded; 14 (23%) of these visits required surgical intervention by a local physician, but no culture material was available for analysis. No patients required referral or hospital admission.

In all, 73 infectious episodes were recorded in 20 of 28 residents in the implicated building, including 43 (59%) skin abscesses, 20 (27%) furuncles, 8 (11%) purulent conjunctivitis, and 2 (3%) external otitis. A mean of seven episodes per month (median 7, range 4–14) peaked in December 1997. The implicated organism was MRSA.

### Epidemiologic Survey

**Figure 1:**
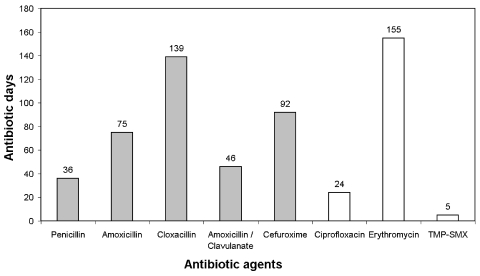
Systemic antibiotic use agents administered to infected residents in Building 15 between March and December 1997. Grids represent beta-lactam agents

The median age of residents in building 15 was 32 years (range 18–45 years), and all residents were male. The mean stay at the institution was 16.3 years ± 6.6 years. We could not identify any known risk factor for MRSA carriage or infection. No residents had been admitted to acute-care hospitals within the 5 years preceding the outbreak, and no contact with known carriers was established. However, 58 courses of oral antibiotics, including amoxicillin, amoxicillin-clavulanate, penicillin, cefuroxime-axetil, cloxacillin, erythromycin, ciprofloxacin, and trimethoprim-sulfamethoxazole, were administered to 16 of 20 (80%) infected residents during a 9-month period, for a total of 572 antibiotic-days (Figure 1). Excess antibiotic consumption was not observed in other buildings.

### Bacteriologic Survey

The first survey was carried out in December 1997 and included 83 cultures, of which 48 were obtained from 28 residents, consisting of 28 (58%) nasal, 10 (21%) perineal, and 10 (21%) exudate cultures. Thirty-four nasal and one exudate cultures were obtained from personnel. Forty of 83 were positive, yielding (48%) grew MRSA or MSSA, for a positive culture rate of 29 (60%) of 48 in residents and 11 (32%) of 35 in personnel. Seventeen (61%) of 28 and 10 (29%) of 34, residents and personnel, respectively, were nasal carriers of *S. aureus*; 16 (95%) and 2 (20%) of the isolates were MRSA. Eighteen (29%) of 62 nasal, 2 (20%) of 10 perineal, and all 11 exudate cultures (including 1 from staff) grew MRSA (a total of 31 isolates).

**Figure 2:**
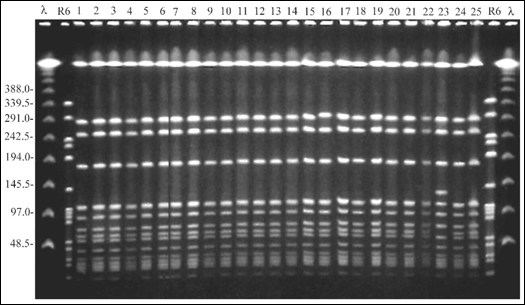
Pulse-field gel electrophoresis of study isolates obtained during the first survey on December 1997 from residents and staff. Lambda ladder and the DNA of the reference *Streptococcus pneumoniae* strain R6, digested by *Sma*I, were used as molecular weight markers. The gel includes 25 representative MRSA isolates. All isolates but one show an indistinguishable banding pattern, thus representing the outbreak strain. Isolate number 23 shows a closely related pattern (one band difference) and is considered to belong to the outbreak strain.

All 31 MRSA isolates were susceptible to ciprofloxacin, gentamicin, fusidic acid, and vancomycin, but 14 (45%) were susceptible to clindamycin and erythromycin. Typing showed that all MRSA isolates belonged to phage-type 29/52/54/95/47/HK2 [52A/79/75/92], an unusual type that had not been isolated before from any patient either in the community or the acute-care setting in southern Israel. All MRSA isolates from both carriers and infected persons yielded an indistinguishable PFGE pattern, except for one isolate that yielded a closely related pattern (one band difference) and was thought to belong to the outbreak strain (Figure 2).

In residents, the 17 nasal carriers had 57 episodes of skin infections during 1997 compared with 16 episodes in 11 noncarriers (p<0.01). At this point, infection control measures were enforced and carriers were randomized to receive intranasal mupirocin (11 cases) or sodium fusidate (14 cases). The same agent was applied topically after drainage.

### Postintervention Survey

The nasal carriage eradication rate was 11 (100%) of 11 for mupirocin and 9 (64%) of 14 for sodium fusidate (p=0.08). The five carriers in whom fusidic acid failed were retreated 2 weeks later with mupirocin and complete eradication was achieved.

In January 1998, a third survey was carried out, including 72 cultures (62 persons), 8 (11%) of which were positive. Of the residents’ cultures, five (62.5%), consisting of two nasal and three exudate cultures, grew MRSA. Three nasal cultures obtained from personnel grew MSSA. One-fifth of the remaining residents and personnel were screened (80 persons), and none were found to be MRSA carriers. No infections were recorded outside building 15 throughout the study period.

Eleven new episodes of furuncles yielding mupirocin-sensitive MRSA were recognized postintervention, involving five residents and one personnel. Six responded to topical mupirocin, while systemic fusidic acid was clinically indicated in five (27 antibiotic days). Since March 1998, no additional episodes were diagnosed in 2 years of follow-up. In March 2000, the nasal culture survey was repeated in the 28 residents. Two (7%) of 28 cultures grew *S. aureus*, MSSA in one resident and MSSA together with MRSA in another. The latter patient had recurrent colonization for 2 years despite repeated eradication efforts, even while the rest of the residents were culture negative.

## Discussion

MRSA is an important pathogen in both acute-care and long-term care facilities (1–7). The complex interaction between patients admitted to chronic- and acute-care facilities is well-known, and the resulting “import” and “export” of MRSA is of great concern (25), making MRSA outbreaks problematic. Defining community-acquired MRSA with accuracy is difficult since widely used criteria seem to reflect “community existence” rather than “community acquisition.” Persistent nasal carriage, which may involve up to 35% of carriers (26,27), can further complicate any attempt to determine the exact time of acquisition.

We describe an outbreak of community-acquired MRSA infections in which the only identified risk factor was a history of heavy exposure to multiple antibiotics, especially beta-lactams, over a relatively short period of time in a population living under closed conditions. The first stage leading to selection of MRSA within MSSA isolates in hospitalized patients is antibiotic treatment (4,28). Such selective pressure permits the growth of a multiresistant bacterial population within a susceptible one, a process that may take <48 hours in approximately 15% of MSSA nasal carriers (4).

Thus, massive antibiotic pressure appears to be an important mechanism for the selection of community-acquired MRSA and its subsequent dissemination when favorable environmental conditions exist, as in the outbreak presented here. Although introduction of an MRSA strain from outside the facility cannot be absolutely ruled out, this possibility is less favorable owing to the epidemiologic features of the population, lack of contact with known carriers or admission to acute-care facilities, lack of risk factors except antibiotic therapy, and the uniqueness of the outbreak strain’s phage type in our country. MRSA emergence in the institution was possible given that contemporary epidemic MRSA clones are actually descendents of old MSSA isolates that had received the *mecA* element and that the evolution of MRSA from of MSSA lineages appears to coincide with selective antibiotic pressure after the introduction of new antibiotics (29).

Skin and soft tissue infections are by far the commonest infections caused by *S. aureus*, and similar to other community-acquired MRSA outbreaks (17–19), recurrent abscesses, or furuncles, or both were the predominant infection in our study. A high prevalence of skin infection has been shown to be a risk factor for persistent nasal carriage of MRSA, augmenting person-to-person transmission (23). Moreover, patients with pathologic skin conditions have a higher risk not only of acquiring skin infections but also of dispersion of infecting strains (27,30,31). Failure to consider MRSA as a cause of such infections may lead to delayed diagnosis and inappropriate therapy, permitting a cycle of disease progression and widespread transmission. Fortunately, no invasive infections occurred as might have been expected in MRSA-colonized patients in nosocomial settings (32). Propagation of the outbreak could be explained by the close and continued physical contact between residents and staff, facilitating transmission, with the high rate of nasal colonization in residents serving as an independent risk factor for MRSA infection (33–35).

Elimination of carriage was achieved by intranasal antibiotics (either mupirocin or fusidic acid); the aim was to control the outbreak and prevent recurrence. We also treated the great majority of infections topically, in addition to bathing with chlorhexidine and appropriate drainage with the intention of maximally reducing systemic antibiotic use.

While mupirocin is a well-accepted eradication regimen (24), the possible emergence of mupirocin resistance during therapy (especially with skin application) should be considered. Fusidic acid, widely used in Israel, has been shown to be a convenient and risk-free method for eradication of nasal *S. aureus* carriage (36), but currently no comparative data evaluate both agents. In our population, only five cases required systemic therapy, according to study indications, and mupirocin resistance was not detected during follow-up. However, persistent nasal MRSA carriage, 2 years after the onset of the outbreak, demonstrates a continued potential threat for both the reemergence of MRSA in the institution and perhaps, dissemination to the community.

Our essential goal was to achieve maximal infection control while maintaining selective pressure at the minimum. Critical factors, which are difficult to achieve in nosocomial settings, allowed us to achieve nearly total eradication of MRSA: 1) only one resident appeared to be a persistent nasal MRSA carrier; 2) all residents were exposed during a specific time frame without mingling between newly admitted or discharged residents; 3) enforcement of strict environmental cleansing and infection control measures; and 4) selective antibiotic pressure owing to minimal use of systemic agents was reduced (only 5 courses).

In conclusion, massive, and perhaps unjustified, systemic antibiotic use in communities, particularly those involving close interaction between members, may permit the emergence of multiresistant bacteria such as MRSA, with a high risk for disease. Implementation of antibiotic control strategies is crucial to prevent the dissemination of MRSA in the community as a whole.

## References

[1] Andersen BM, Bergh K, Steinbakk M, Syversen G, Magnaes B, Dalen H, A Norwegian nosocomial outbreak of methicillin-resistant *Staphylococcus aureus* resistant to fusidic acid and susceptible to other antistaphylococcal agents. J Hosp Infect. 1999;41:123–32. 10.1016/S0195-6701(99)90049-X 10063474

[2] Goetz MB, Mulligan ME, Kwok R, O’Brien H, Caballes C, Garcia JP. Management and epidemiologic analyses of an outbreak due to methicillin-resistant *Staphylococcus aureus.* Am J Med. 1992;92:607–14. 10.1016/0002-9343(92)90778-A 1605142

[3] Barrett FF, McGehee RF Jr, Finland M. Methicillin resistant *Staphylococcus aureus* at Boston City Hospital. N Engl J Med. 1968;279:441–8. 4232865 10.1056/NEJM196808292790901

[4] Thompson RL, Cabezudo I, Wenzel RP. Epidemiology of nosocomial infections caused by methicillin-resistant *Staphylococcus aureus.* Ann Intern Med. 1982;97:309–17. 7114627 10.7326/0003-4819-97-3-309

[5] Terpenning MS, Bradley SF, Wan JY, Chenoweth CE, Jorgensen KA, Kauffman CA. Colonization and infection with antibiotic resistant bacteria in a long term care facility. J Am Geriatr Soc. 1994;42:1062–9. 7930330 10.1111/j.1532-5415.1994.tb06210.x

[6] O’Toole RD, Drew WL, Dahlgren BJ, Beaty HN. An outbreak of methicillin-resistant *Staphylococcus aureus* infection: observation in hospital and nursing home. JAMA. 1970;213:257–63. 10.1001/jama.213.2.257 5467891

[7] Storch GA, Radecliff JL, Meyer PL, Hinrichs JH. Methicillin-resistant *Staphylococcus aureus* in a nursing home. Infect Control. 1987;8:24–9. 3643889 10.1017/s0195941700066947

[8] Boyce JM. Are the epidemiology and microbiology of methicillin-resistant S*taphylococcus aureus* changing? JAMA. 1998;279:623–4. 10.1001/jama.279.8.623 9486761

[9] Akram J, Glatt AE. True community-acquired methicillin-resistant *Staphylococcus aureus* bacteremia. Infect Control Hosp Epidemiol. 1998;19:106–7. 9510108 10.1086/647775

[10] Herold BC, Immergluck LC, Maranan MC, Lauderdale DS, Gaskin RE, Boyle-Vavra S, Community-acquired methicillin-resistant *Staphylococcus aureus* in children with no identified predisposing risk. JAMA. 1998;279:593–8. 10.1001/jama.279.8.593 9486753

[11] Moreno F, Crisp C, Jorgensen JH, Patterson JE. Methicillin-resistant *Staphylococcus aureus* as a community organism. Clin Infect Dis. 1995;21:1308–12. 8589164 10.1093/clinids/21.5.1308

[12] Boyce JM. Methicillin-resistant *Staphylococcus aureus*: detection, epidemiology and control measures. Infect Dis Clin North Am. 1989;3:901–13. 2687368

[13] Fraise AP, Mitchell K, O’Brien S, Wise R. Methicillin-resistant *Staphylococcus aureus* in the community. Lancet. 1995;346:850. 10.1016/S0140-6736(95)91670-9 7674784

[14] Levine DP, Cushing RD, Jui J, Brown WJ. Community-acquired methicillin- resistant *Staphylococcus aureus* endocarditis in the Detroit Medical Center. Ann Intern Med. 1982;97:330–8. 7114630 10.7326/0003-4819-97-3-330

[15] Steinberg JP, Clark CC, Hackman BO. Nosocomial and community-acquired *Staphylococcus aureus* bacteremias from 1980 to 1993: impact of intravascular devices and methicillin-resistance. Clin Infect Dis. 1996;23:255–9. 8842259 10.1093/clinids/23.2.255

[16] Gottlieb RD, Shah MK, Perlman DC, Kimmelman CP. Community-acquired methicillin-resistant *Staphylococcus aureus* infections in otolaryngology. Otolaryngol Head Neck Surg. 1992;107:434–7. 1408231 10.1177/019459989210700316

[17] Saravolatz LD, Markowitz N, Arking L, Pohlod D, Fischer E. Methicillin-resistant *Staphylococcus aureus*. Epidemiologic observations during a community-acquired outbreak. Ann Intern Med. 1982;96:11–6. 7053683 10.7326/0003-4819-96-1-11

[18] Lindenmayer JM, Schoenfeld S, O’Grady R, Carney JK. Methicillin-resistant *Staphylococcus aureus* in a high school wrestling team and the surrounding community. Arch Intern Med. 1998;158:895–9. 10.1001/archinte.158.8.895 9570176

[19] Maguire GP, Arthur AD, Boustead PJ, Dwyer B, Currie BJ. Emerging epidemic of community-acquired methicillin-resistant *Staphylococcus aureus* in the Northern Territory. Med J Aust. 1996;164:721–3. 8668078 10.5694/j.1326-5377.1996.tb122270.x

[20] Beaujean DJ, Weersink AJ, Blok HE, Frenay HM, Verhoef J. Determining risk factors for methicillin-resistant *Staphylococcus aureus* carriage after discharge from hospital. J Hosp Infect. 1999;42:213–8. 10.1053/jhin.1999.0585 10439994

[21] Hiramatsu K, Kihara H, Yokota T. Analysis of borderline-resistant strains of methicillin-resistant *Staphylococcus aureus* using polymerase chain reaction. Microbiol Immunol. 1992;36:445–53. 1513261 10.1111/j.1348-0421.1992.tb02043.x

[22] Roberts RB, de Lencastre A, Eisner W, Severina EP, Shopsin B, Kreiswirth BN, Molecular epidemiology of methicillin-resistant *Staphylococcus aureus* in 12 New York hospitals. MRSA Collaborative Study Group. J Infect Dis. 1998;178:164–71. 9652436 10.1086/515610

[23] Tenover FC, Arbeit RD, Goering RV, Mickelsen PA, Murray BE, Persing DH, Interpreting chromosomal DNA restriction patterns produced by pulse-field gel electrophoresis: criteria for bacterial strain typing. J Clin Microbiol. 1995;33:2233–9. 7494007 10.1128/jcm.33.9.2233-2239.1995 PMC228385

[24] Wenzel RP, Reagan DR, Bertino JS Jr, Baron EJ, Arias K. Methicillin-resistant *Staphylococcus aureus* outbreak: a consensus panel’s definition and management guidelines. Am J Infect Control. 1998;26:102–10. 10.1016/S0196-6553(98)80029-1 9584803

[25] Kauffman CA, Bradley SF, Terpenning MS. Methicillin-resistant *Staphylococcus aureus* in long-term facilities. Infect Control Hosp Epidemiol. 1990;11:600–3. 2124234 10.1086/646102

[26] Vandenbergh MF, Yzerman EP, Van Belkum A, Boelens HA, Sijmons M, Verbrugh HA. Follow-up of *Staphylococcus aureus* nasal carriage after 8 years. Redefining the persistent carrier state. J Clin Microbiol. 1999;37:3133–40. 10488166 10.1128/jcm.37.10.3133-3140.1999 PMC85511

[27] Mulligan ME, Murray-Leisure KA, Ribner BS, Standiford HC, John JF, Korvick JA, Methicillin-resistant *Staphylococcus aureus*: a consensus review of the microbiology, pathogenesis and epidemiology with implications for prevention and management. Am J Med. 1993;94:313–28. 10.1016/0002-9343(93)90063-U 8452155

[28] McGowan JE Jr. Antimicrobial resistance in hospital organisms and its relation to antibiotic use. Rev Infect Dis. 1983;5:1033–48. 6318289 10.1093/clinids/5.6.1033

[29] Crisostomo MI, Westh H, Tomasz A, Chung M, Oliviera DC, de Lencastre H. The evolution of methicillin resistance in *Staphylococcus aureus*: Similarity of genetic backgrounds in historically early methicillin-susceptible and–resistant isolates and contemporary epidemic clones. Proc Natl Acad Sci U S A. 2001;98:9865–70. 10.1073/pnas.161272898 11481426 PMC55544

[30] Vandenbergh MF, Verbrugh HA. Carriage of *Staphylococcus aureus*: epidemiology and clinical relevance. J Lab Clin Med. 1999;133:525–34. 10.1016/S0022-2143(99)90181-6 10360626

[31] Solberg CO. A study of carriers of *Staphylococcus aureus* with special regard to quantitative bacterial estimations. Acta Med Scand. 1965;436(Suppl):1–96. 5318930

[32] Pujol M, Pena C, Pallares R, Ariza J, Ayats J, Dominguez MA, Nosocomial *Staphylococcal aureus* bacteremia in nasal carriers of methicillin-resistant and methicillin susceptible strain. Am J Med. 1996;100:509–16. 10.1016/S0002-9343(96)00014-9 8644762

[33] Nichols RL. Surgical wound infection. Am J Med. 1991;91:S54–64. 10.1016/0002-9343(91)90344-W 1928192

[34] Luzar MA. Exit-site infection in continuous ambulatory peritoneal dialysis: a review. Perit Dial Int. 1991;11:333–40. 1751600

[35] Lye WC, Leong SO, Lee EJ. Methicillin-resistant *Staphylococcus aureus* nasal carriage and infections in CAPD. Kidney Int. 1993;43:1357–62. 10.1038/ki.1993.191 8315950

[36] Hedstrom SA. Treatment and prevention of recurrent Staphylococcal furunculosis: clinical and bacteriological follow-up. Scand J Infect Dis. 1985;17:55–8. 10.3109/00365548509070420 3992206

